# Google Street View Images as Predictors of Patient Health Outcomes, 2017–2019

**DOI:** 10.3390/bdcc6010015

**Published:** 2022-01-27

**Authors:** Quynh C. Nguyen, Tom Belnap, Pallavi Dwivedi, Amir Hossein Nazem Deligani, Abhinav Kumar, Dapeng Li, Ross Whitaker, Jessica Keralis, Heran Mane, Xiaohe Yue, Thu T. Nguyen, Tolga Tasdizen, Kim D. Brunisholz

**Affiliations:** 1 Department of Epidemiology and Biostatistics, School of Public Health, University of Maryland, College Park, MD 20742, USA; 2 Healthcare Delivery Institute, Intermountain Healthcare, Salt Lake City, UT 84107, USA; 3 School of Computing, Scientific Computing and Imaging Institute, University of Utah, Salt Lake City, UT 84112, USA; 4 Department of Computer Science and Engineering, Michigan State University, East Lansing, MI 48824, USA; 5 Department of Geography and Geospatial Sciences, South Dakota State University, Brookings, SD 57007, USA; 6 Department of Electrical and Computer Engineering, University of Utah, Salt Lake City, UT 84112, USA

**Keywords:** Google Street View, built environment, neighborhood characteristics, patient health, social determinants of health, computer vision

## Abstract

Collecting neighborhood data can both be time- and resource-intensive, especially across broad geographies. In this study, we leveraged 1.4 million publicly available Google Street View (GSV) images from Utah to construct indicators of the neighborhood built environment and evaluate their associations with 2017–2019 health outcomes of approximately one-third of the population living in Utah. The use of electronic medical records allows for the assessment of associations between neighborhood characteristics and individual-level health outcomes while controlling for predisposing factors, which distinguishes this study from previous GSV studies that were ecological in nature. Among 938,085 adult patients, we found that individuals living in communities in the highest tertiles of green streets and non-single-family homes have 10–27% lower diabetes, uncontrolled diabetes, hypertension, and obesity, but higher substance use disorders—controlling for age, White race, Hispanic ethnicity, religion, marital status, health insurance, and area deprivation index. Conversely, the presence of visible utility wires overhead was associated with 5–10% more diabetes, uncontrolled diabetes, hypertension, obesity, and substance use disorders. Our study found that non-single-family and green streets were related to a lower prevalence of chronic conditions, while visible utility wires and single-lane roads were connected with a higher burden of chronic conditions. These contextual characteristics can better help healthcare organizations understand the drivers of their patients’ health by further considering patients’ residential environments, which present both risks and resources.

## 1. Introduction

The importance of the built environment as a determinant of health is well established in the literature [1]. The quality of neighborhood conditions has been shown to influence the prevalence of obesity, diabetes, and risk of mortality [2,3]. Certain built environment features can facilitate accessibility, which in turn can influence physical and mental health. For example, roads and public transportation can improve access to nutrition and healthcare facilities, while built environment features such as parks and trails can help promote physical activities [4–9]. Previous research has reported the influence of neighborhood features such as presence of roadways, buildings, access to public transportation, green spaces, and walkability on both physical and mental health outcomes [10–13]. Interconnected streets and mixed land use in urban neighborhoods have been linked to increased physical activity [14]. In our previous research, we found that built environment features at the ZIP code level such as green streets, crosswalks, and commercial buildings were associated with a lower prevalence of individual-level obesity and diabetes [15].

The aim of this study is to leverage publicly available Google Street View (GSV) images to construct indicators of the neighborhood-built environment for the state of Utah. Google Street View (GSV) image data mitigates some of the limitations of traditional sources of neighborhood data used for individual-level health outcome analysis. Traditionally, administrative data and neighborhood surveys have served as sources of data on neighborhood conditions and provided insights regarding how residents perceive their neighborhood environment. While these data sources provide assessments of neighborhood features that are considered important for health by residents, they are self-reported data and are subject to social desirability bias and same-source bias (for example, neighborhood conditions and health outcomes might be correlated because the health influences the exposure assessment by the individual) [16,17]. In-person audits are another source of data on the built environment, but they can be expensive and time consuming. As an alternative, Google Street View (GSV) images can serve as a reliable and cost-effective data source to capture features of neighborhood environments [18]. Virtual audits using GSV images has been found it to be consistent with field assessments [18–20] and able to effectively discern built environment features such as commercial buildings, crosswalks, and highways [2,15].

Recent progress in computer vision, an interdisciplinary field using artificial intelligence, has advanced studies that identify, process, and analyze video and image data to derive meaningful information. To analyze GSV images, we used trained Visual Geometry Group (VGG-19 model) deep convolutional networks [21,22]. Earlier image recognition models such as Fisher Vectors [23] used handcrafted features, while the recent models [24–26] are all based on deep learning. Earlier deep learning models such as AlexNet [21], GoogleNet [27], and VGG-Net [22] used sequential Convolution Neural Networks (CNN) architectures and were limited to a few layers, while the recent ones [25,28] are variations of residual CNNs. Despite being very deep, the variations of residual CNNs are trainable because of the introduction of the batch normalization layer [29]. More recent methods [26] have removed the batch normalization to make these networks compact. CNNs assume translational equivariance of the image data [30] and, therefore, only handle short-range dependencies. Another class of architectures called Transformers [24] removes the translational equivariance assumption and allows long-range dependencies with soft attention.

In this study, we created neighborhood indicators derived from GSV images analyzed by CNNs in order to examine the effects of neighborhood environments on individual-level health outcomes of about one-third of people living in Utah by leveraging electronic medical records from one of the largest healthcare providers in Utah, Intermountain Healthcare. The use of electronic medical records allows for the assessment of associations between neighborhood characteristics and individual-level health outcomes while controlling for predisposing factors, which distinguishes this study from previous GSV studies that were ecological in nature. Outcomes examined include obesity, diabetes, high blood pressure, and substance use disorders. Findings from this study can help inform clinical practice regarding neighborhood characteristics that are connected with patient health outcomes.

## 2. Materials and Methods

### 2.1. Study Setting and Population

Patient data were acquired from 2017 to 2019 from Intermountain Healthcare, a Utah based integrated not-for-profit healthcare system which includes 24 hospitals with 2900 licensed beds and 215 owned or supported clinics. Annually, Intermountain Healthcare provides 495,000 emergency department (ED) visits, 136,000 inpatient admissions, and 160,000 inpatient and ambulatory surgeries. Patients included in the dataset were those who were 18 years and older, had a medical visit from 2017–2019, and were Utah residents (*n* = 1,433,316). Analyses were restricted to those with non-missing data on covariates and health outcomes and stratified by urbanicity. The majority of patients served by Intermountain lived in urban areas, and hence, these are reported in the main tables (n = 938,085 with non-missing data on covariates and health outcomes). In the Appendixes A and B, we present data on n = 53,414 participants who lived in rural areas in Utah.

### 2.2. Study Measurement

#### Individual-Level Characteristics

From Intermountain Healthcare, we obtained individual-level health outcomes for eligible patients to study the prevalence of type 2 diabetes, high blood pressure, and obesity (body mass index ≥30 kg/m^2^). Type 2 diabetes and hypertension were defined according to the National Committee for Quality Assurance (NCQA) Healthcare Effectiveness Data and Information Set (HEDIS) specifications [31]. Type 2 diabetes specifications require only one of the following to be met along with a diagnosis code of diabetes (ICD-9 code: 250): (a) two outpatient encounters on different dates of service; (b) one acute inpatient encounter; (c) one emergency department visit; or (d) patients who were dispensed insulin or hypoglycemic/anti-hyperglycemics on an ambulatory basis. Individuals were identified with hypertension if they had one outpatient encounter with a hypertension diagnosis code during the study period. Other outcomes included type 2 diabetes control (HbA1c ≥7%) and substance use disorders (includes any of the following: alcohol, opioid, cannabis, sedative, hypnotics, anxiolytics, cocaine, other stimulates including caffeine, hallucinogens, inhalants, other psychoactive substances and multiple drug use). Following HEDIS specifications for these outcomes, patients with evidence of end-stage renal disease, kidney transplant, pregnancy, or admission to a non-acute inpatient facility (e.g., skilled nursing facility) were excluded.

Sociodemographic characteristics included age (continuous), race (White: yes/no), ethnicity (Hispanic: yes/no), marital status (married: yes/no), religious affiliation (any/none), insurance (yes/no), and area deprivation index (ADI). The ADI is a geographic area-based measure of the disadvantaged position of residents relative to the society [32]. The ADI was calculated for the state of Utah using a measure developed by Singh et al. [33] based upon 17 US Census measures associated with mortality, including living conditions, income, unemployment, and education. Census measures were based on the 2013 American Community Survey published by the US Census Bureau.

### 2.3. Google Street View Image Data

#### 2.3.1. Google Street View Image Data Collection

GSV image data was collected using GSV Image API. We focused on all primary and secondary roads in Utah, mainly street intersections and other locations along road segments. We sampled locations at a 50 m interval, and for each set of coordinates, we gathered GSV images from four directions (facing west, east, north, and south) to best describe the neighborhood quality and environment. In total, 1,394,442 images from Utah were obtained in November 2019.

#### 2.3.2. Built Environment Indicators

The selected indicators include building type (the presence of any non-single-family detached house: yes/no), roads with a single lane (yes/no), crosswalk presence (yes/no), street greenness (at least 30% of the image consisted of trees and landscaping: yes/no), and the presence of visible utility wires overhead (yes/no). To select the indicators for this study, we considered built neighborhood characteristics deemed important in the literature as well as which indicators would be suitable for computer vision models. For health outcomes, the literature has identified three indicators as being essential for the study: neighborhood walkability [34–36], neighborhood disorder [37–39], and mixed land use [40–42]. The presence of crosswalks has traditionally been used to indicate the walkability of an area as well as to measure health outcomes and behaviors. Sidewalks were also considered, but because of their high prevalence in urban areas, they offer less variability.

We constructed a measure of mixed land use because its impact on travel behavior and resource accessibility is well studied. In single-use residential areas, individuals may need to rely on using motorized transportation to get to their destinations. Conversely, areas that include residential, commercial, and leisure destinations may offer more opportunities for walking or biking, and physical activities and health-promoting resources may also be more accessible [43]. An indicator for non-single-family home was created to distinguish between entirely residential areas with only detached homes and areas with various building types, including businesses, schools, apartments, and cultural venues. Single-lane roads were selected to serve as an indicator of lower urban development to distinguish between areas with higher capacity for cars and people versus areas with less capacity.

Regarding street greenness, we found that in our dataset, street landscaping was prevalent in the images, and we strove to create an indicator that could distinguish between ample versus sparse street landscaping. Thus, we chose a cut point of 30% such that an image was classified as being a green street if approximately 30% of the image was street trees or street landscaping.

Furthermore, we also identified visible wires from the images. Although research on visible wires is a burgeoning area of study, more literature can be found abroad. In Rio de Janeiro, not only are visible wires unattractive, they are also a fire and electrocution hazard [44]. In the United States, visible wires have a similar visual impact. We selected visible wires as an indicator to further the literature, and we explore their associations with health outcomes. The undesirable aesthetics of visible wires, as well as their health risk, could deter health-promoting activities (by discouraging walking) and could have negative mental health implications (by increasing stress).

#### 2.3.3. Image Data Processing

Convolutional Neural Networks (ConvNets) [20,22,28] achieve state-of-the-art accuracy for many computer vision tasks, including object recognition, object detection, and scene labeling. ImageNet [45], a large-scale visual database, includes 1000 categories (e.g., “balloon”, “motorcycle”, “strawberry”) and over one million image samples. A ConvNet model “pre-trained” based on ImageNet can be “fine-tuned” (known as optimizing configurations that control the model learning process to achieve better performance) using a smaller training dataset from the target task. This tuning process helps deliver high performance and does not require a potentially very large training dataset and computational resources to train the original ConvNet model.

18,700 images, dating from December 2016 to February 2017 were manually labeled by the principal investigator and three graduate research assistants. The distinctive labels of the neighborhood characteristics included presence of crosswalk, building type (single-family detached house vs. other), visible utility wires, single-lane roads, and street greenness (trees and landscaping comprised at least 30% of the image—yes/no). The locations of the images included a national sample, as well as images from Charleston, WV, USA, Salt Lake City, UT, USA, and Chicago, IL, USA, and were selected to include a diverse range of neighborhood characteristics within the US. 80% of the labeled images were randomly selected for training and validation of the computer visual models and 20% of the remaining dataset was used for testing the computer vision models’ performance. Hyperparameters were tried on a trial and error basis and tuned to optimize accuracy on the validation set. After choosing the hyperparameters, we trained each model architecture multiple times. It is important to understand that the neural network training process is stochastic (meaning randomness is involved) even when using the same initialization and training set; therefore, we required multiple training runs to check the mean and standard deviation of the error. We did not use the test set during any step of the training process; it remained unobserved until we finished selecting the best model using the training set and validation set. Then, we assessed the best model performance by using the test set.

To process the GSV images, we first resized all the images to be 224 × 224. A standard deep convolutional neural network architecture, Visual Geometry Group VGG-19 [22] in TensorFlow [46], was used to train the model with sigmoid cross entropy with logits as the loss function. The weights of the network were initialized from the pre-trained ImageNet model. A batch size of 20 was used along with Adam optimizer. The learning rate was set to start with 1 × 10^−4^, and training took 20 epochs. The model in the last epoch was considered the final model. The accuracy of the classification tasks (agreement between manual annotations and computer vision predictions) was high: street greenness (88.70%), presence of crosswalks (97.20%), non-single family home (82.35%), single-lane roads (88.41%), and visible utility wires (83.00%).

#### 2.3.4. Neighborhood Definitions

Census tracts were chosen as the neighborhood unit because of their relatively uniform population characteristics, economic status, and living conditions [47]. In general, census tracts range from populations of 1200 to 8000, with an optimum size of 4000. To arrive at the neighborhood indicators, we processed street imagery and then combined information on all street imagery within a census tract to arrive at census tract-level summaries (e.g., percentage of images in a census tract that contain a crosswalk). We derived aggregated measures for green streets, crosswalks, non-single-family homes, single-lane roads, and visible wires and created tertiles for all the built environment indicators based on these measures. Tertiles were utilized to allow for nonlinearities in the relationship between built environment characteristics and health outcomes.

### 2.4. Statistical Analyses

The data on neighborhood features were merged with the individual-level health outcomes and sociodemographic data for patients. We implemented log Poisson regression models to examine the association between tertiles of built-environment indicators and individual chronic disease prevalence after adjusting for individual-level sociodemographic characteristics. Outcomes examined included diabetes prevalence, uncontrolled diabetes, high blood pressure, obesity, and substance use disorder. A variety of health outcomes were chosen to determine the range with which GSV images can predict patient health outcomes. Main predictors included tertiles for green space, crosswalk, non-single-family homes, single-lane roads, and visible utility wires. Health outcomes were compared for patients living in neighborhoods in the third tertile (and second tertile) of built environment characteristics vs. the first tertile (lowest level). Models were also adjusted for age, race, ethnicity, religious affiliation, health insurance status, and ADI. Separate models were run for each health outcome. Statistical significance was assessed with an alpha level of 0.05. SAS 9.4 software was utilized for analyses (SAS Institute Inc., Cary, NC, USA).

## 3. Results

**Figure 1.**
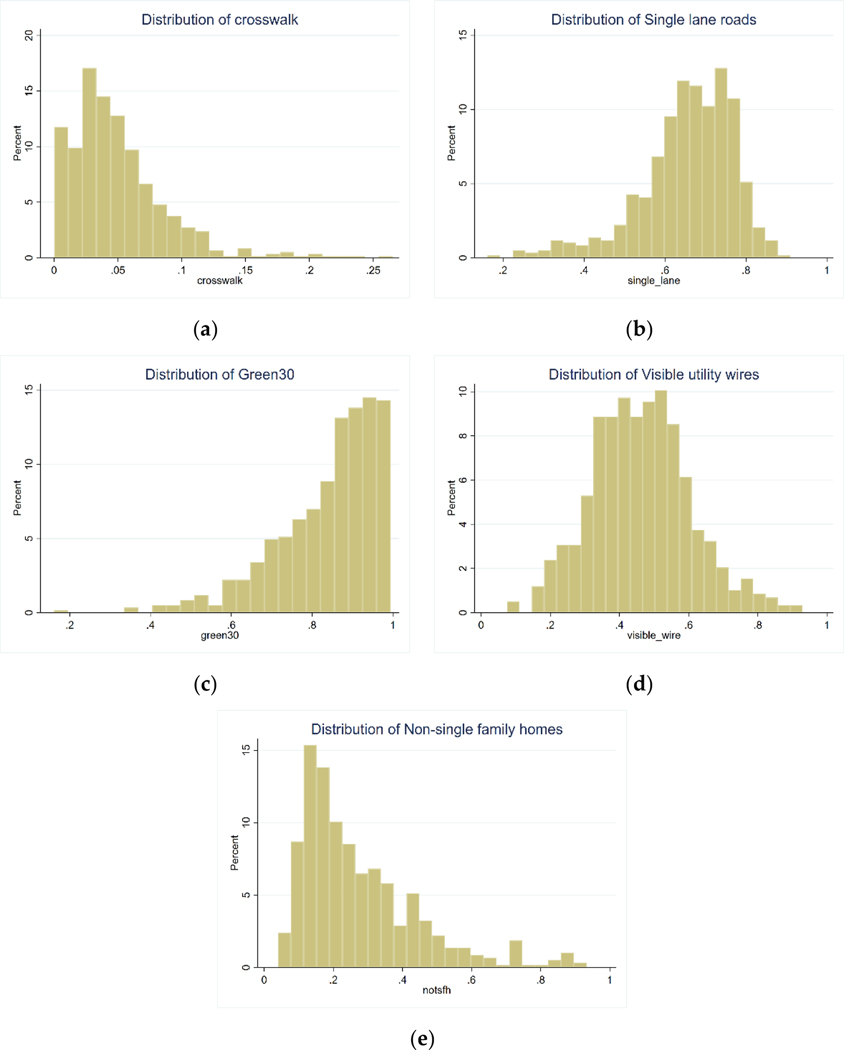
Distribution of built environment characteristics in Utah. Histograms are presented for the following built environment characteristics: (**a**) presence of crosswalk, (**b**) single-lane road, (**c**) green street, (**d**) visible utility wires overhead, and (**e**) buildings other than single-family homes. The Y-axis represents the percent of census tracts in the dataset, and the X-axis represents the percent of a given built environment characteristic among images for an area. For example, for single-lane roads, only 5% of census tracts (X-axis) have 80% of its images containing single-lane roads (Y-axis).

Table 1 summarizes descriptive statistics of our study population and their census tract neighborhood environment derived from GSV images. The mean age was 47 years with about 57% being female, 58% being married, 11% being Hispanic/Latinx, and 5% being non-White. About 28% were self-pay (uninsured), and 68% reported a religious affiliation. The prevalence of obesity was 47%, and the prevalence of diabetes was 6%. Figure 1 displays the distribution of the GSV-derived built environment characteristics. Single-lane roads and visible utility wires were unimodal and relatively common characteristics. Street greenness was right-skewed, with most census tracts having prevalence of 60% and above. Non-single-family homes were left-skewed, with the majority of census tracts having prevalence of less than 40%. Crosswalks, the rarest of the built environment characteristics, were also left-skewed, with the majority of census tracts having prevalence of less than 10%.

**Figure 2.**
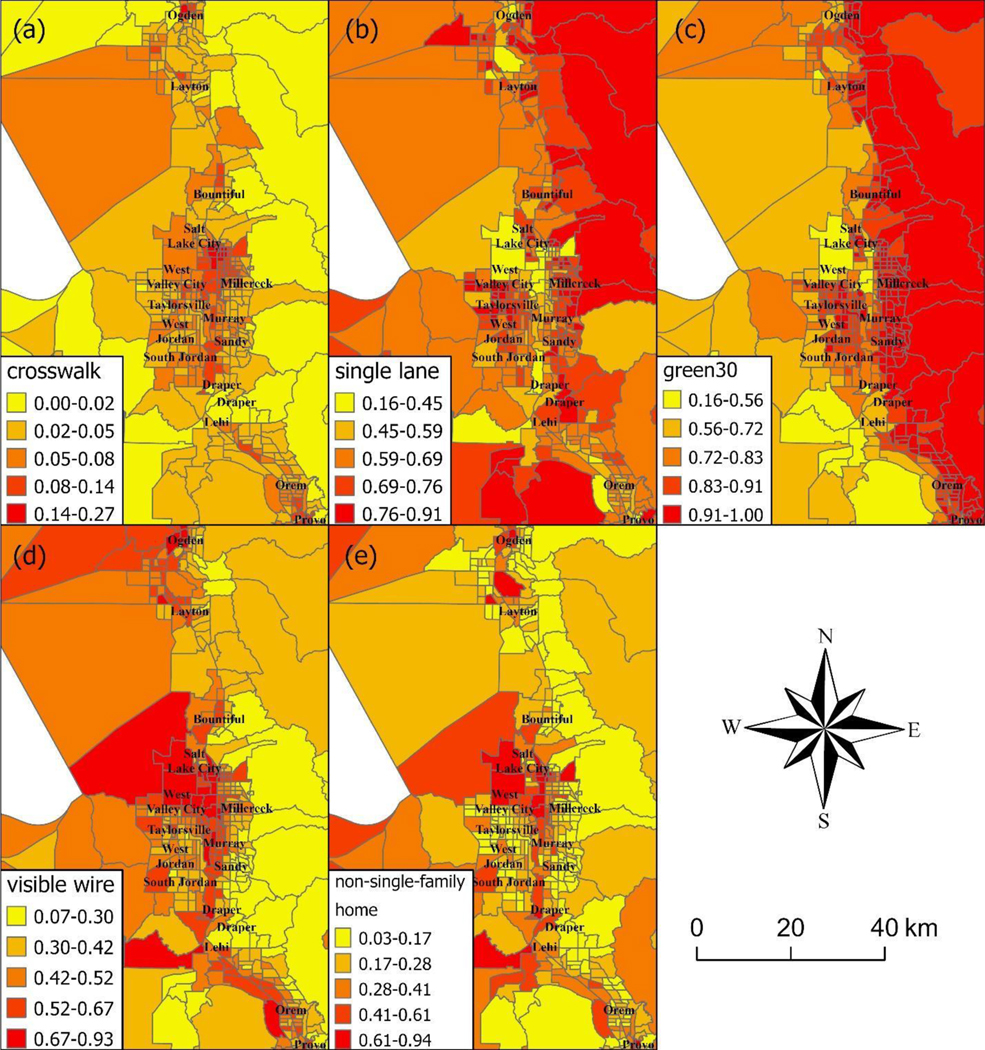
Geographical distribution of built environment characteristics in Utah. Figure presents the spatial distribution of Google Street View (GSV)-derived built environment characteristics across the Wasatch Front, which contains the major cities of Salt Lake City, West Valley City, Provo, West Jordan, Layton, and Ogden, where the majority of Utah residents live. The numbers in the legend specify categories of percentages of built environment characteristics among the GSV images for that area. Darker colors signify higher percentages of a given built environment feature. Built environment features mapped include (**a**) presence of crosswalk, (**b**) single-lane road, (**c**) green street, (**d**) visible utility wires overhead, and (**e**) buildings other than single-family homes.

Figure 2 presents the spatial distribution the GSV-derived built environment features across the Wasatch Front, which contains the major cities of Salt Lake City, West Valley City, Provo, West Jordan, Layton, and Ogden, where the majority of Utah residents live. Single-lane roads were concentrated in areas such as the eastern part of Salt Lake City, Bountiful, West Valley City, Millcreek, Sandy, and Draper City (Utah County). Street greenness was concentrated throughout eastern Utah. Crosswalks were present only in a few locations (e.g., Salt Lake City, South Salt Lake, Murray, Ogden, and Provo) in the urban core. Visible utility wires and non-single-family homes were present in the urban core (e.g., Salt Lake City and South Salt Lake) and also dispersed throughout western Utah.

Table 2 presents the estimated prevalence ratios and 95% CIs for all the examined associations between tertiles of built environment indicators and individual health outcomes, controlling for individual age, White race, Hispanic ethnicity, religious affiliation, marital status, and lack of health insurance. In all models, GSV-derived built environment variables were statistically significantly associated with health outcomes, with green space and non-single-family homes being protective of negative outcomes. Comparing the third tertile with the first tertile, non-single-family homes were associated with a 17% lower prevalence of diabetes (95% CI: 0.81–0.85), 14% lower prevalence of uncontrolled diabetes (95% CI: 0.82–0.89), 27% lower prevalence of hypertension (95% CI: 0.67–0.80), and 11% lower prevalence of obesity (95% CI: 0.88–0.90). Green streets were associated with decreased diabetes (PR: 0.90; 95% CI: 0.88–0.92)), uncontrolled diabetes (PR: 0.89; 95% CI: 0.86–0.92), hypertension (PR: 0.84; 95% CI: 0.78–0.90), and obesity (PR: 0.90; 95% CI: 0.89–0.91). However, both green streets and non-single-family homes were tied to an increased prevalence of substance use disorders, 17% (95% CI: 1.13–1.21) and 12% (95% CI: 1.08–1.17), respectively.

An increase in visible wires was associated with a higher prevalence of all adverse outcomes, although not all comparisons for the 3rd and 2nd tertiles reached statistical significance. More visible wires were associated with 9–10% higher prevalence of diabetes and uncontrolled diabetes and a 4–5% increase in obesity. Visible utility wires were also linked to increased hypertension and substance use. Surprisingly, more crosswalks (mainly concentrated in Utah’s urban core) were associated with 7–9% increased prevalence of hypertension and only weakly associated with other health outcomes. Single-lane roads were generally not associated with health outcomes, except for a slight increase in diabetes (Table 2). Patterns are similar in rural areas, but associations were more attenuated, and the statistical power was less given the fewer number of Intermountain patients living in rural areas (*n* = 53,414; Table A1).

Individual characteristics were also associated with health outcomes, and all tended to be statistically significant except for English as a primary language, which had little effect and was removed from the final model. White race was associated with better health outcomes, including a lower prevalence of diabetes, uncontrolled diabetes, hypertension, and obesity (Table 2). Hispanic ethnicity was associated with increased diabetes, uncontrolled diabetes, and obesity. Religious affiliation was associated with more diabetes, more uncontrolled diabetes, and obesity, but it was protective of hypertension. Marital status (married) was positively associated with hypertension.

To examine whether individual-level disadvantages were associated with certain built environments, we implemented log Poisson models to examine predictors of uninsured status among Intermountain patients. Uninsured patients were less likely to live in neighborhoods with green streets and to live in neighborhoods with fewer or no single-family homes. They were more likely to live in neighborhoods with visible utility wires overhead and were slightly more likely to live in neighborhoods with single-lane roads and crosswalks (Table 3).

We examined associations between GSV-derived built environment indicators and other census tract-level characteristics. The percentage of non-Hispanics Blacks was related to less exposure to green space and single-lane roads and more exposure to visible utility wires and non-single-family homes. Median household income was related to more green space and fewer visible utility wires and non-single-family homes (Table 4).

## 4. Discussion

While a large body of literature has connected neighborhood built environment characteristics with an array of health outcomes, neighborhood data beyond sociodemographic characteristics can be time consuming and expensive to gather; thus, it is largely unavailable for large areas of the country. In this study, we leverage high-resolution GSV images from across the state of Utah to construct indicators of the built environment. Then, we examined whether these built environment characteristics were associated with patient health outcomes. Working with Intermountain Healthcare, a major provider of care in Utah, we examined health patterns for close to 1 million patients. Our study found that non-single-family homes (an indicator of mixed land use and urban development) and green streets were related to a lower prevalence of chronic conditions. Conversely, visible utility wires and single-lane roads were connected with a higher burden of chronic conditions. This aligns with previous studies conducted at the census tract, county, and state levels that have found similar associations for non-single-family homes, single-lane roads, and visible utility wires [3,48]. For example, a previous state-level GSV study has linked non-single-family homes to decreased diabetes and premature mortality and increased physical activity [48]. Additionally, previous county-level analyses found that urban development was related to lower chronic disease burden and decreased premature mortality [2]. However, those studies were ecological in nature, while the current study is one of the few utilizing individual-level data.

In this study with individual-level patient data, we found that crosswalks (an indicator of walkability) were related to worse health outcomes, which is counter to our study hypotheses. Previous research involving the 500 Cities Project found mixed results with crosswalks [3]. Areas that were relatively dense with crosswalks (third tertile) had lower obesity, diabetes, and physical inactivity, but areas with “medium” amounts of crosswalks (second tertile) experienced higher rates of obesity, diabetes, and physical inactivity compared areas with the fewest crosswalks (first tertile). While an increase in crosswalks is likely to facilitate walking and physical activity, an increase in area-level crime would deter walking. Thus, these complex relationships between crosswalks and health outcomes might be influenced by factors such as neighborhood crime, which were not considered in this study. The distribution of crosswalks was more left-skewed and rarer than any other variable (Figure 1). Crosswalks might also be more likely placed in core urban centers where the most disadvantaged individuals might live (Figure 2). In addition, individuals without health insurance were slightly more likely to live in areas with more crosswalks (Table 2).

We additionally found that green streets and non-single-family homes were related to a higher prevalence of substance use disorders. Street landscaping and the presence of other building types besides single detached family homes might indicate higher urbanicity. The landscape of Utah, with its sandy deserts, red rocks, and deep canyons, generally has less natural greenness, which might mean that areas with more green landscaping denote higher urban development. In previous GSV analyses, we found that higher urban development was related to more excessive drinking [2].

This study also examined predictors of built environment by health insurance status. Uninsured patients were more likely to live in areas with visible utility wires, single-lane roads, and crosswalks. Uninsured patients were less likely to live in areas with green streets and non-single homes. In one of our previous studies, we found that greater county-level economic disadvantage was associated with a lower prevalence of non-single-family homes and visible wires at the county level after adjusting for violent crime rate, age, race/ethnicity, percentage of population not proficient in English, and ratio of population to primary care providers [49].

### Study Strengths and Limitations

This is among the few studies examining GSV-derived predictors of individual-level outcomes, controlling for individual-level predisposing characteristics. Previous studies with GSV images have utilized ecological frameworks [48]: for instance, county-level built environment predictors of county health outcomes [49]. In partnership with one of the largest healthcare providers in Utah, in this study, we included close to one-third of the population in Utah. We find that GSV-derived built environment characteristics were linked with an array of important health outcomes. Study findings suggest that structuring neighborhoods to locate amenities where people live and adding street landscaping could reduce chronic disease and improve health. Conversely, physical disorder could increase health risks through potential mechanisms such as decreased perception of safety and social cohesion, decreased physical activity, and poorer mental health status [38,39,50,51].

Nonetheless, our study is subject to limitations. While we utilized data from one of the main healthcare providers in Utah, there may be differences between the composition of patients at Intermountain and residents of Utah as a whole. For example, females are slightly over-represented, comprising 54.4% of the Intermountain sample versus 49.6% of the Utah population according to census estimates [52]. Additionally, a higher proportion of Intermountain patients are White versus the overall population in Utah (95.4% vs. 90.6%) [52]. Future studies incorporating patient health records from multiple healthcare providers and from other states can further help to investigate potential health impacts of neighborhood environments in different populations. Additionally, future studies may wish to employ longitudinal designs to examine whether changes in neighborhood environments predict changes in health outcomes. Google Street View API now allows for the capture of historical images. Difficulties for a computer vision model might include changes in season, zoom, and angle of images taken across various time points, with computer vision models needing to be robust to these perturbations to correctly quantify real changes in neighborhood environments. Additional complexities might include unequal time gaps across image updates (e.g., 1 year, 2 years) depending on Google Street View’s update schedule for particular geographical areas. Urban areas also tend to have more frequent image updates than rural areas. Collecting more images across longer time spans and measuring changes in health outcomes can provide valuable information about the impact of changing neighborhood environments on changes in health outcomes.

## 5. Conclusions

We leveraged GSV images and computer vision to characterize neighborhood environments. Nonetheless, it is important to note that this study does not include other distinct neighborhood constructs that could have health implications such as air quality and pollution, and perceived neighborhood safety and area walkability [53]. Although computer vision is a useful tool that helps identify, process, and analyze images, it is often limited to features that are larger in size. Moreover, since the training datasets for the computer vision are manually annotated, the number of features that could be studied are limited. Thus, unlike onsite neighborhood inventories that can potentially include hundreds of neighborhood features, we focused on a select few neighborhood features whose connection to health outcomes has been theoretically or empirically established in the literature. These contextual characteristics can better help healthcare organizations understand the drivers of their patients’ health by further considering patients’ residential environments, which present both risks and resources.

## Figures and Tables

**Table 1.** Descriptive statistics of study population, Utah, 2019.

	N^a^	Mean (Standard Deviation)/% (95% CI)

Individual-level covariates		
Age (years)	1,433,316	46.53 (19.03)
% Female	1,433,316	54.36% (54.28–54.45)
% Married	1,069,207	58.06% (57.98–58.14)
% White	1,346,584	95.39% (95.35–95.42)
% Hispanic ethnicity	1,357,627	10.83% (10.78–10.88)
% Uninsured	1,433,316	28.39% (28.31–28.46)
% Religious affiliation	1,069,207	68.17% (68.08–68.25)
Area deprivation index	1,433,298	97.51 (18.61)
Health outcomes		
% Obesity	1,374,731	47.28% (47.19–47.36)
% Diabetes	1,433,316	5.88% (5.84–5.92)
Hemoglobin A1c (%)	1,433,316	9.23% (9.18–9.28)
% Hypertension	1,433,316	0.69% (0.68–0.71)
Google Street View (Census tract)		
Green street	1,394,442	83.76 (12.68)
Crosswalk	1,394,442	4.95 (3.82)
Non-single-family home^b^	1,394,442	27.53 (17.24)
Single-lane road	1,394,442	65.56 (11.65)
Visible utility wires	1,394,442	46.19 (14.36)

a N reports the number of individuals with covariate and health outcome data. For GSV images, N reports the number of images analyzed.

b Non-single-family home = presence of a building that is not a single-family home (e.g., schools, grocery stores and other businesses denoting mixed land use).

**Table 2.** Associations between built environment characteristics and individual-level health outcomes.

	Diabetes	Uncontrolled Diabetes	Hypertension	Obesity	Substance Use Disorder

	Prevalence Ratio (95% CI) ^b^	Prevalence Ratio (95% CI) ^b^	Prevalence Ratio (95% CI) ^b^	Prevalence Ratio (95% CI) ^b^	Prevalence Ratio (95% CI) ^b^

GSV indicators					
Green streets, 3rd tertile	0.90 (0.88, 0.92) *	0.89 (0.86, 0.92) *	0.84 (0.78, 0.90) *	0.90 (0.89, 0.91) *	1.17 (1.13, 1.21) *
Green streets, 2nd tertile	0.99 (0.97, 1.01)	0.98 (0.95, 1.01)	0.98 (0.93, 1.05)	0.98 (0.97, 0.98) *	1.06 (1.03, 1.09) *
Crosswalks, 3rd tertile	1.02 (1.00, 1.05) *	1.01 (0.98, 1.04)	1.07 (1.00, 1.14) *	1.01 (1.00, 1.02) *	1.00 (0.97, 1.03)
Crosswalks, 2nd tertile	1.01 (0.99, 1.03)	1.00 (0.98, 1.03)	1.09 (1.02, 1.16) *	1.02 (1.01, 1.02) *	0.99 (0.96, 1.02)
Non-single-family home, 3rd tertile	0.83 (0.81, 0.85) *	0.86 (0.82, 0.89) *	0.73 (0.67, 0.80) *	0.89 (0.88, 0.90) *	1.12 (1.08, 1.17) *
Non-single-family home, 2nd tertile	0.91 (0.89, 0.93) *	0.91 (0.88, 0.94) *	0.89 (0.83, 0.96) *	0.95 (0.95, 0.96) *	1.03 (0.99, 1.06)
Single-lane roads, 3rd tertile	1.02 (0.99, 1.04)	1.00 (0.97, 1.04)	0.94 (0.87, 1.01)	1.00 (0.99, 1.01)	0.98 (0.95, 1.02)
Single-lane roads, 2nd tertile	1.03 (1.01, 1.05) *	1.01 (0.99, 1.04)	0.98 (0.92, 1.04)	1.00 (1.00, 1.01)	0.97 (0.94, 1.00)
Visible wires, 3rd tertile	1.09 (1.06, 1.11) *	1.10 (1.06, 1.14) *	1.05 (0.97, 1.14)	1.04 (1.03, 1.06) *	1.05 (1.01, 1.09) *
Visible wires, 2nd tertile	1.09 (1.07, 1.12) *	1.10 (1.07, 1.13) *	1.08 (1.01, 1.16) *	1.05 (1.04, 1.05) *	0.99 (0.96, 1.02)
Covariates					
Age (years)	1.04 (1.04, 1.04) *	1.03 (1.03, 1.03) *	1.01 (1.01, 1.01) *	1.01 (1.01, 1.01) *	1.00 (1.00, 1.00)
White race	0.60 (0.58, 0.62) *	0.53 (0.51, 0.55) *	0.80 (0.72, 0.90) *	0.93 (0.91, 0.94) *	1.16 (1.10, 1.22) *
Hispanic ethnicity	1.15 (1.12, 1.18) *	1.34 (1.30, 1.39) *	0.96 (0.88, 1.05)	1.08 (1.07, 1.09) *	0.68 (0.65, 0.70) *
Any religion	1.21 (1.19, 1.23) *	1.18 (1.15, 1.21) *	0.86 (0.82, 0.91) *	1.07 (1.06, 1.07) *	0.65 (0.64, 0.67) *
Married	1.09 (1.07, 1.11) *	1.03 (1.01, 1.05) *	1.40 (1.33, 1.48) *	1.12 (1.11, 1.13) *	0.40 (0.39, 0.41) *
Uninsured	1.60 (1.57, 1.63) *	1.73 (1.69, 1.77) *	1.11 (1.05, 1.17) *	1.10 (1.09, 1.11) *	2.38 (2.33, 2.44) *
Area deprivation index	1.01 (1.01, 1.01) *	1.01 (1.01, 1.01) *	1.00 (1.00, 1.00) *	1.01 (1.01, 1.01) *	1.01 (1.01, 1.01) *

For GSV indicators, reference category is 1st tertile.

b Adjusted Log Poisson regression controlled for the following covariates: age, White race, Hispanic ethnicity, any religious affiliation, marital status, self-pay status for health insurance, area deprivation index. *N* = 938,085

* *p* < 0.05.

**Table 3.** Predicting uninsured status with neighborhood- and individual-level characteristics.

	Prevalence Ratio (95% CI)

GSV indicators	
Green streets, 3rd tertile	0.89 (0.87, 0.92) *
Green streets, 2nd tertile	1.01 (0.99, 1.03)
Crosswalks, 3rd tertile	1.08 (1.05, 1.10) *
Crosswalks, 2nd tertile	1.06 (1.04, 1.08) *
Non-single-family home, 3rd tertile	0.85 (0.83, 0.87) *
Non-single-family home, 2nd tertile	0.88 (0.86, 0.90) *
Single-lane roads, 3rd tertile	1.06 (1.03, 1.08) *
Single-lane roads, 2nd tertile	1.04 (1.01, 1.06) *
Visible wires, 3rd tertile	1.32 (1.29, 1.35) *
Visible wires, 2nd tertile	1.23 (1.20, 1.25) *
Covariates	
Age (years)	1.04 (1.04, 1.04) *
White race	0.57 (0.55, 0.59) *
Hispanic ethnicity	1.33 (1.29, 1.36) *
Any religion	1.23 (1.21, 1.25) *
Married	1.03 (1.01, 1.05) *

Adjusted Poisson regression controlled for all variables listed simultaneously, *N* = 938,085

* *p* < 0.05. For Google Street View indicators, the reference category is the 1st tertile.

**Table 4.** Associations between census tract sociodemographics and Google Street View-derived built environment characteristics, census tract level.

		Built Environment Indicators			

Census Tract Characteristics ^a^	Green Space	Crosswalk	Non-Single-Family Home	Single-Lane Roads	Visible Wire

	Prevalence (95% CI)	Prevalence (95% CI)	Prevalence (95% CI)	Prevalence (95% CI)	Prevalence (95% CI)

% non-Hispanic Black	−43.68 (−60.61, −26.74) *	13.84 (9.08, 18.61) *	70.67 (48.88, 92.45) *	−67.12 (−84.09, −50.16) *	51.00 (32.75, 69.24) *
% Hispanic	0.16 (−2.00, 2.32)	−0.38 (−0.99, 0.23)	−3.50 (−6.28, −0.72) *	4.01 (1.85, 6.18) *	2.54 (0.21, 4.86) *
% Unemployed	1.72 (0.07, 3.36) *	0.34 (−0.13, 0.80)	0.83 (−1.29, 2.95)	−0.57 (−2.22, 1.08)	−0.26 (−2.04, 1.52)
Median household income	7.46 (5.75, 9.17) *	−0.70 (−1.18, −0.22) *	−11.59 (−13.79, −9.39) *	5.68 (3.97, 7.40) *	−10.55 (−12.39, −8.70) *
Household size	−2.96 (−3.89, −2.04) *	−0.76 (−1.02, −0.50) *	−2.56 (−3.75, −1.36) *	−0.33 (−1.26, 0.60)	−0.09 (−1.09, 0.91)
Population density	5.90 (5.00, 6.80) *	1.57 (1.32, 1.83) *	−5.65 (−6.81, −4.50) *	0.95 (0.05, 1.85) *	−2.69 (−3.66, −1.73) *

All predictor variables are standardized to have a mean of 0 and standard deviation of 1.

* *p* < 0.05; *N* = 586 census tracts in Utah.

**Figure A1.**
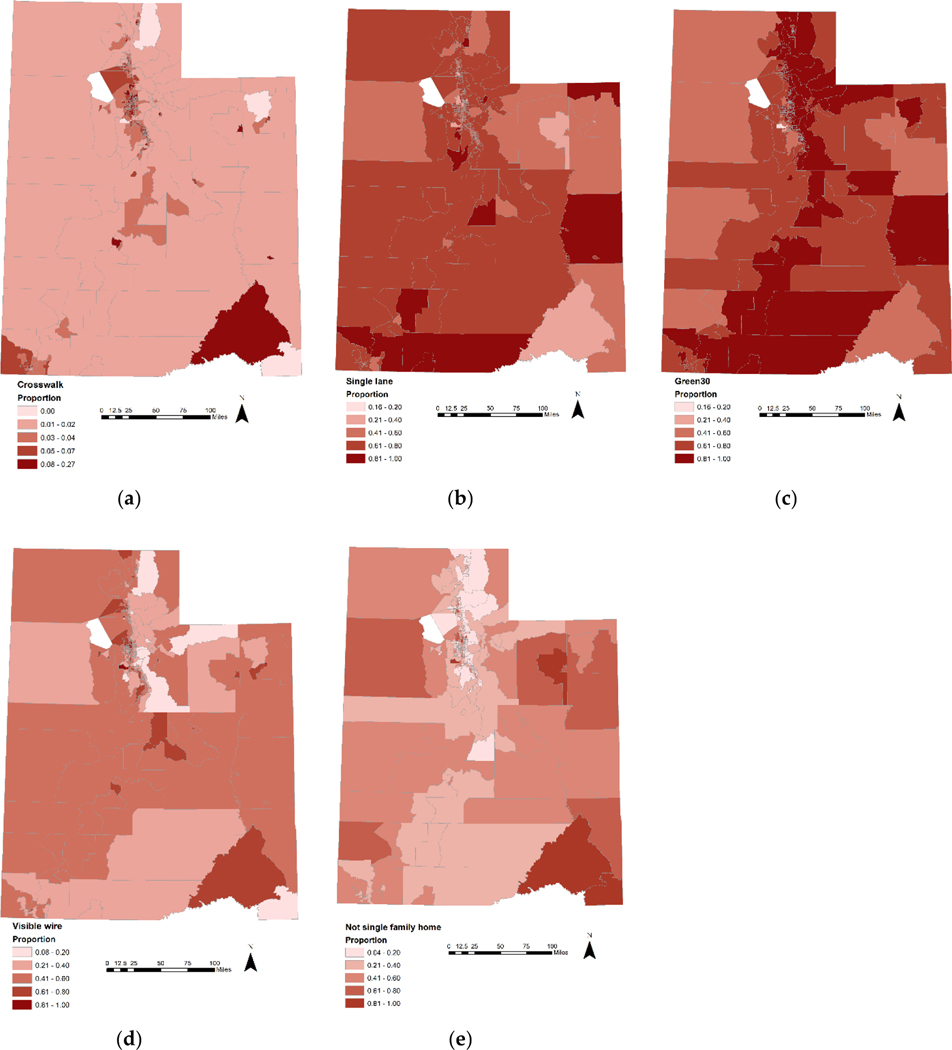
Geographical distribution of built environment characteristics in Utah. The figure presents the spatial distribution of Google Street View-derived built environment characteristics across Utah. The numbers in the legend specify categories of percentages of built environment characteristic among the GSV images for that area. Darker colors signify higher percentages of a given built environment feature. Built environment features mapped include (**a**) presence of crosswalk, (**b**) single-lane road, (**c**) green street, (**d**) visible utility wires overhead, and (**e**) buildings other than single-family homes.

## Appendix B

**Table A1.** Associations between built environment characteristics and individual-level health outcomes among non-urban areas in Utah.

	Diabetes	Uncontrolled Diabetes	Hypertension	Obesity	Substance Use Disorder

	Prevalence Ratio (95% CI)	Prevalence Ratio (95% CI)	Prevalence Ratio (95% CI)	Prevalence Ratio (95% CI)	Prevalence Ratio (95% CI)

Google Street View indicators					
Green streets, 3rd tertile	1.19 (0.96, 1.48)	1.03 (0.92, 1.15)	1.03 (0.54, 1.99)	0.90 (0.83, 0.98) *	0.98 (0.70, 1.38)
Green streets, 2nd tertile	1.03 (0.95, 1.12)	1.32 (0.96, 1.80)	0.78 (0.59, 1.04)	0.97 (0.94, 1.00) *	0.98 (0.86, 1.11)
Crosswalks, 3rd tertile	1.06 (0.47, 2.38)	1.06 (0.90, 1.24)	1.26 (0.17, 9.17)	0.99 (0.73, 1.33)	1.41 (0.58, 3.44)
Crosswalks, 2nd tertile	1.05 (0.93, 1.18)	1.15 (0.43, 3.10)	1.35 (0.96, 1.90)	1.01 (0.97, 1.06)	1.18 (1.00, 1.39) *
Non-single-family home, 3rd tertile	0.87 (0.70, 1.08)	0.99 (0.72, 1.36)	1.04 (0.54, 2.00)	0.93 (0.85, 1.01)	0.88 (0.63, 1.22)
Non-single-family home, 2nd tertile	1.02 (0.84, 1.24)	1.04 (0.78, 1.39)	1.12 (0.62, 2.02)	0.98 (0.90, 1.06)	0.85 (0.63, 1.16)
Single-lane roads, 3rd tertile	1.06 (0.94, 1.19)	0.96 (0.82, 1.12)	1.07 (0.76, 1.52)	1.02 (0.98, 1.07)	1.08 (0.91, 1.27)
Single-lane roads, 2nd tertile	1.08 (0.95, 1.22)	1.02 (0.87, 1.20)	1.03 (0.71, 1.49)	1.02 (0.97, 1.07)	1.13 (0.95, 1.34)
Visible wires, 3rd tertile	1.26 (1.12, 1.43) *	1.19 (1.01, 1.40) *	1.01 (0.69, 1.49)	1.10 (1.04, 1.15) *	1.14 (0.95, 1.37)
Visible wires, 2nd tertile	1.17 (1.04, 1.32) *	1.19 (1.00, 1.41) *	0.81 (0.55, 1.17)	1.05 (1.01, 1.10) *	1.01 (0.84, 1.20)
Covariates					
Age (years)	1.01 (1.01, 1.01) *	1.03 (1.02, 1.03) *	1.00 (1.00, 1.01) *	1.01 (1.01, 1.01) *	1.00 (1.00, 1.01) *
White race	0.60 (0.58, 0.62) *	0.57 (0.43, 0.76) *	0.84 (0.39, 1.78)	0.83 (0.76, 0.92) *	0.77 (0.57, 1.04)
Hispanic ethnicity	1.15 (1.12, 1.18) *	1.46 (1.19, 1.78) *	0.64 (0.34, 1.21)	1.07 (1.01, 1.14) *	0.61 (0.47, 0.80) *
Any religion	1.21 (1.19, 1.23) *	1.39 (1.24, 1.55) *	1.02 (0.81, 1.30)	1.10 (1.07, 1.14) *	0.59 (0.54, 0.66) *
Married	1.09 (1.07, 1.11) *	0.94 (0.85, 1.03)	1.50 (1.16, 1.93) *	1.16 (1.12, 1.19) *	0.45 (0.41, 0.50) *
Uninsured	1.60 (1.57, 1.63) *	1.98 (1.79, 2.18) *	1.28 (1.00, 1.62) *	1.12 (1.08, 1.15) *	2.60 (2.35, 2.87) *
Area deprivation index	1.01 (1.01, 1.01) *	1.02 (1.01, 1.02) *	1.00 (0.99, 1.01)	1.01 (1.01, 1.01) *	1.00 (1.00, 1.01) *

Adjusted Log Poisson regression controlled for the following covariates: age, white race, Hispanic ethnicity, any religion, marital status, health insurance status, area deprivation index. *N* = 53,414.
